# Patient Characteristics Associated with Free Nipple Graft Reduction Mammoplasty

**DOI:** 10.7759/cureus.9063

**Published:** 2020-07-08

**Authors:** Andrea Y Lo, Roy P Yu, Anjali Raghuram, Phillip Khim, Sarah Wang, Haig L Manoukian, Maxwell Johnson, Wesley Schooler, Joseph Carey, Ketan M Patel, Regina Y Baker, Alex K Wong

**Affiliations:** 1 Plastic Surgery, Keck School of Medicine of the University of Southern California, Los Angeles, USA; 2 Plastic and Reconstructive Surgery, Keck School of Medicine of the University of Southern California, Los Angeles, USA

**Keywords:** reduction mammoplasty, free nipple graft, gigantomastia

## Abstract

Purpose: Surgical approaches for reduction mammoplasty most commonly incorporate a parenchymal vascular pedicle. For patients with larger breasts where pedicle viability may be compromised due to excessive length, the free nipple graft (FNG) technique provides a safe alternative. Criteria for whether a patient should undergo a FNG remains controversial due to variable reports in the literature with small sample sizes and inherent surgeon-dependent bias. To address this, we sought to investigate perioperative factors associated with performing FNGs at our institution in order to better elucidate specific indications for this surgery.

Methods: A retrospective chart review was performed for 323 patients who underwent a reduction mammoplasty from August 2009 to July 2019 at Keck Hospital and LAC+USC Medical Center. Data regarding patient demographics, comorbidities, pre-operative breast characteristics, and post-operative complications were extracted. Studentʼs t-test, Fisherʼs exact test, and logistic regression were performed in R.

Results: Of 323 patients, 15 received an FNG. Independent variables analyzed included: age, body mass index (BMI), obesity, smoking, diabetes, hypertension, surgical indication, sternal notch-to-nipple length, nipple-to-inframammary fold length, and weight of breast specimens removed. BMI, obesity, gigantomastia, and weight of specimen resected were significantly associated with use of the FNG (p < 0.001, p < 0.05, p < 0.0001, p < 0.0001, respectively). Regression analysis revealed that patients who had an average of more than 1500 g of tissue removed from each breast were 1.41 (95% CI: 1.17-1.71, p<0.001) times more likely to undergo an FNG procedure than those who had less than 1500 g of tissue removed. Demographic data and breast characteristics, such as notch-to-nipple length and nipple-to-inframammary fold length, were not significantly associated.

Conclusion: Total weight of the breast specimens removed and BMI were significantly associated with the FNG technique. Removing more than 1500 g gof total breast tissue was also significantly correlated. These findings may guide surgeons during the decision-making process of when to use an FNG.

## Introduction

Women with excessive breast hypertrophy or macromastia suffer from a number of complaints, including chronic neck, shoulder, and back pain, intertrigo, impaired self-esteem, and compromised physical functioning [1]. Conservative management with physical therapy, weight loss, and use of more appropriately fitting brassieres does not adequately address patient symptoms [2]. As a result, reduction mammoplasty, or surgical resection of breast parenchymal tissue, is an effective procedure that is commonly performed by plastic surgeons. Reduction mammoplasty has high reported rates of decreased patient pain scores, satisfaction with postoperative breast shape, and improved overall quality of life [3,4].

Proposed techniques for reduction mammoplasty differ in terms of skin incision placement, tissue resection patterns, and retention of blood supply to the remaining breast tissue and areolar complex [2]. However, these techniques are limited in terms of their applicability for significant breast hypertrophy, or gigantomastia. Gigantomastia requires removal of greater than 1500 g of breast tissue from a single breast and poses unique reconstructive challenges [2]. Traditionally, for patients who need removal of greater than 1000 g of breast tissue, plastic surgeons have advocated against the use of pedicle techniques. Given the questionable pedicle viability in this patient population, these techniques are thought to increase the risk of nipple areolar complex (NAC) ischemia and subsequent necrosis [5].

In order to minimize nipple viability complications with breast reduction, the free nipple graft (FNG) technique is performed for patients with either gigantomastia or marked breast ptosis [6]. One such approach involves the incorporation of a de-epithelialized inferior pedicle buried as an autologous implant, followed by pedicle shortening and transfer to the NAC as a free graft [7]. This technique confers flexibility and ease in shaping the breast for reduction and ensuring improved post-surgical shape. Moreover, the FNG approach can be decided upon preoperatively or intraoperatively with improved pedicle visualization. Nevertheless, the FNG approach faces criticism for increased operative time, loss of NAC sensation, poor nipple projection, loss of lactation ability, and uneven pigmentation from partial epidermolysis [5,6,8].

Recognizing the need for nuanced decision-making to tailor breast reduction approaches for a clinically diverse population, it is useful to consider perioperative patient factors that can guide surgical selection of the FNG approach. To date, there are few patient studies that have clearly attempted to characterize patient, breast, and procedural elements that would favor the use of free nipple grafting. Thus, the aim of this study was to elucidate specific indicators for performing an FNG. We performed a retrospective chart review of 323 patients who have undergone reduction mammoplasty between August 2009 and July 2019 at Keck Hospital and LAC+USC Medical Center. Our assessment of patients who received the FNG approach in their reconstruction offers informative data for more comprehensively evaluating and counseling patients in their operative planning for the management of breast hypertrophy.

## Materials and methods

This study was approved by the Institutional Review Board. A multi-institution, multi-surgeon retrospective chart review was performed on all patients who underwent a reduction mammoplasty between August 2009 to July 2019 at Keck Hospital and LAC+USC Medical Center. No patients were excluded from the analysis.

Patient demographics, comorbidities, breast characteristics, surgery information, and postsurgical outcomes were extracted from patient charts. Univariate analyses were performed in order to compare the patient demographic, comorbidities, and breast characteristic data between patients who underwent an FNG operation and those who did not. Data regarding post-operative complications was also extracted and compared between the two groups. Continuous variables are expressed as mean and were analyzed using an unpaired Student’s t-test. Categorical variables are expressed as proportions and were analyzed using Fisher’s exact test. All statistical analyses were performed using R version 3.6.1.

Multivariable logistic regression was used to identify predictive factors associated with performing an FNG procedure. The initial multivariable logistic regression model included all variables from the univariate analyses that were associated with an FNG at a significance level of p<0.25 and all variables that were cited as potential predictors in literature. Multicollinearity was assessed among possible predictors by calculating the tetrachoric/polychoric correlation coefficient and variance inflation factor (VIF) for each variable. A VIF of 10 or greater indicates a high degree of intercorrelation; there was no evidence of multicollinearity among the possible predictors. From the results of the initial multivariable logistic regression model, a significance level of 0.25 was required to allow a variable into the base model. Variables were then removed and added in a stepwise fashion until a final model with no indicator variable eligible for entry into the model met of model entry. After a main effects model was finalized, a potential interaction between obesity and weight of breast specimen removed was tested by adding the interaction term along with any main effects that were removed in a previous step to the model. An interaction significance level of p<0.15 was considered significant. Results are reported as odds ratios (OR) and 95% confidence intervals (CI).

## Results

**Figure 1:**
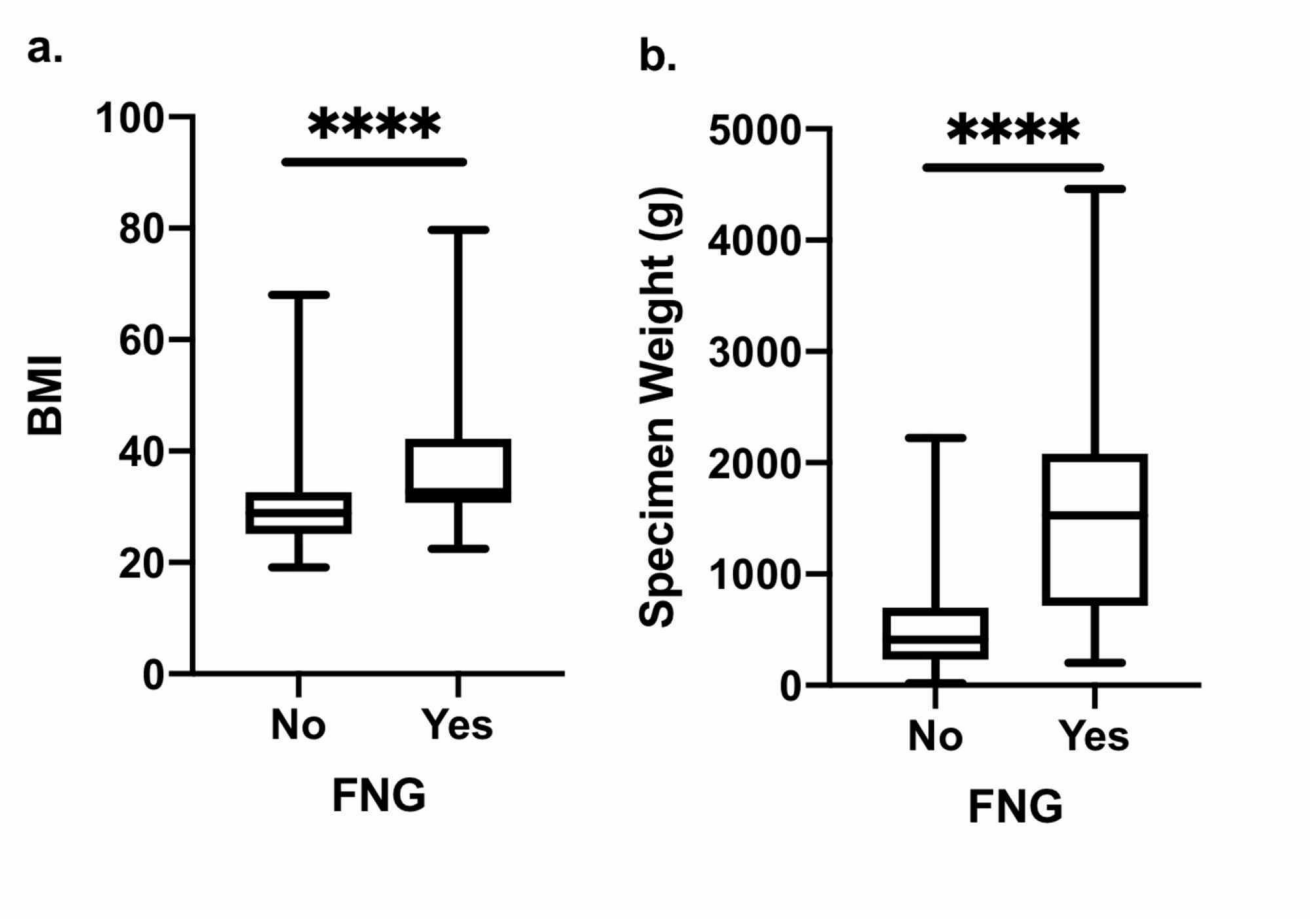
Box and whisker plots displaying distribution of body mass indexes (BMI). (a.) and individual breast specimen weights (b.) between breast reduction mammoplasty patients who did and did not receive a free nipple graft (FNG) surgery (****p<0.0001).

Of the 323 patients included in the study who underwent a reduction mammoplasty, an FNG was performed in 15 patients (4.6%). Patient demographics, comorbidities, and breast characteristics data of all patients included in the study are presented in Table 1. The mean age for those who had an FNG procedure done was 55.1 years while the mean age of patients who did not undergo an FNG procedure was 50.2 years. Compared to patients who did not undergo an FNG procedure, those who did had a significantly higher body mass index (BMI) (37.6 vs. 29.5, p<0.0001), and in turn, exhibited a higher proportion of obesity (78.6% vs. 46.0%, p<0.05). In addition, patients who underwent an FNG also displayed a greater proportion of those who had “gigantomastia” listed as an indication for surgery in the operative note (20.0% vs. 0%, p<0.0001). Of note, only a total of three out of 323 patients in the cohort were diagnosed with gigantomastia by their physician. In addition to BMI, the breast specimen weight was significantly higher in FNG patients than in non-FNG patients (1600 g vs. 498 g, p<0.0001). The distribution of this data in our patient population is highlighted as box and whisker plots in Figure 1. Pre-operative breast measurements, such as nipple-to-IMF distance and sternal notch-to-nipple distance, did not significantly differ between the two groups.

**Table 1:** Comparison of demographic factors, comorbidities, resection weight, and pre-operative measurements between reduction mammoplasty patients who did and did not receive a free nipple graft (FNG) procedure. Data is reported as (mean ± standard deviation) when possible. Significant differences are shown with p values based off of a student’s T test. *Data in these categories are represented as per individual breast.

Demographic Factor	Free Nipple Graft (FNG)		
	No (n=323)	Yes (n=15)	P-value
Age (yrs)	50.2 ± 15.0	55.1 ± 11.2	0.12
BMI (kg/m^2^)	29.5 ± 5.98	37.6 ± 14.0	< 0.0001
Comorbidity (%):			
Obesity	46.0% (121)	78.6% (11)	0.05
Smoking	13.3% (51)	15.8% (2)	1.00
Diabetes	13.3% (27)	8.4% (2)	0.37
Hypertension	26.7% (61)	18.9% (4)	0.49
Surgical Indication			
Macromastia	58.5% (189)	53.3% (8)	0.79
Gigantomastia	0.% (0)	20% (3)	<0.0001
Breast Cancer	36.5% (118)	26.4% (4)	0.59
Ruptured Implant(s)	0.9% (3)	0.% (0)	1.00
Capsular Contracture	0.9% (3)	0.% (0)	1.00
Other	2.5% (8)	0.% (0)	1.00
Specimen Resected:*			
Weight (g)	498 ± 367	1600 ± 1076	< 0.0001
Range (g)	20 – 2222	200 – 4460	
Nipple to IMF:*			
Distance (mm)	89.0 ± 62.6	94.8 ± 98.6	0.74
Range (mm)	5.50 – 290	11.0 – 230	
Sternal Notch to Nipple:*			
Distance (mm)	210 ± 136	202 ± 228	0.82
Range (mm)	12.0 – 450	21.5 – 620	

Dogmatic plastic surgery teaching has implied that the viability of pedicled breast parenchymal flaps, regardless of whether they are based inferiorly, medially, laterally, or superiorly, is dependent on pedicle length however in the literature this remains controversial [5,9,10]. Using our data set, we set out to address the importance of pedicle length as well as other potential predictive variables based on a review of the relevant literature. Using a model entry criterion of p<0.25, the potential set of predictor variables included age, obesity, degree of ptosis, average nipple-to-IMF distance, average sternal notch-to-nipple distance, and weight category of breast specimen removed. After logistic regression analysis with stepwise selection, three independent predictors of performing an FNG were included in the final model (Table 2). The final set of predictors included weight category of breast specimen removed, obesity, and degree of ptosis. Based on the final logistic regression model, patients who required more than an average of 1500 g of tissue to be removed from each breast were 1.41 (95% CI: 1.17-1.71, p<0.001) times more likely to undergo an FNG procedure than those who have less than 1500 g of tissue removed.

**Table 2:** Final multivariate model (n=323). P-values comparing the cohorts were obtained using multiple logistic regression analysis. *The specimen weight of each breast was included and analyzed using Student’s t-test. **No patients were reported to have a degree of ptosis = 1.

Variable	OR (95% CI)	P-value
Weight of Breast Specimen Removed, g*		
<500g		
500-1000g	1.072 (0.997-1.153)	0.06
1000-1500g	1.013 (0.892-1.150)	0.84
>1500g	1.416 (1.172-1.712)	<0.001
Obesity		
No		
Yes	0.985 (0.917-1.059)	0.68
Degree of Ptosis**		
2		
3	0.993 (0.934-1.056)	0.862

Lastly, post-operative complication rates between the two groups are seen in Table 3. The total post-operative complication rate was 19.8% in patients who did not receive an FNG and 33.3% in patients who did receive an FNG. However, the difference was not statistically significant and neither of the individual complications seen in Table 3 were statistically significant either.

**Table 3:** Complication rates in breast reduction mammoplasty patients who did and did not receive a free nipple graft (FNG) surgery.

Complication	Free Nipple Graft (FNG)		
	No (n=323)	Yes (n=15)	P-value
Total post-operative complication rate (%)	19.8%	33.3%	0.20
Wound complication			
Dehiscence	8.99% (29)	6.67% (1)	1.00
Infection	1.86% (6)	6.67% (1)	0.27
Nonhealing	1.55% (5)	6.67% (1)	0.24
Necrotic tissue	1.24% (4)	0.% (0)	1.00
Hematoma	2.79% (9)	13.3% (2)	0.08
Seroma	0.929% (3)	0.% (0)	1.00
Cosmetic result			
Poor cosmetic result	4.64% (15)	6.67% (1)	0.52
Asymmetry	6.81% (22)	6.67% (1)	1.00

## Discussion

Reduction mammoplasty is a common plastic surgery procedure with the goal of reducing breast weight while creating an aesthetically pleasing breast and maintaining nipple sensation. There are multiple methods to achieve this, with the inferior pedicle technique being the most common [11]. However, for patients with heavily hypertrophied breasts, the FNG technique is considered due to concern for pedicle viability [5]. This procedure is not ideal as it compromises nipple sensation, and thus, is a last resort option amongst many plastic surgeons.

To date, specific pre-surgical indicators for an FNG procedure are not clear. Previous studies have shown that pedicle length and therefore the status of nipple perfusion are determinant factors for converting to FNG intraoperatively [5,9]. The use of laser Doppler intraoperatively and postoperatively can be useful in monitoring perfusion and determining whether conversion to FNG is necessary to prevent NAC necrosis [5,12]. Yet, one study has shown that while a laser Doppler can serve as a valuable supplement, a surgeon’s clinical expertise and judgement were the main factors that decided whether an FNG should be performed [12].

Some studies have hinted that pre-operative breast measurements such as sternal notch to nipple or inframammary fold (IMF) to nipple distances could help predict pedicle length and whether a FNG should be performed [5,9,10]. One study suggests that a suprasternal notch to nipple distance > 30 cm can help identify patients at risk for vascular complications, specifically venous complications to the NAC, which can result in tissue necrosis [10]. Another study also suggested that suprasternal notch to nipple distance was significantly longer in FNG patients [13]. In our study, the sternal notch to nipple distance was 210 mm and 202 mm for patients who did not receive an FNG and those who did, respectively (Table 1). The distribution of these measurements was wide with standard deviations of 136 mm and 226 mm, for the two patient populations respectively. Additionally, sternal notch to nipple distance and the IMF to nipple distance did not differ significantly between the two groups and did not correlate with whether an FNG was performed (Tables 1-2). Post-operative complication rates did not differ significantly between the two groups, indicating that the decision to use a pedicle technique did not significantly compromise the surgical result (Table 3).

According to our study, breast resection weights >1500 g correlated significantly with the FNG procedure, with these patients being 1.42 times more likely to receive an FNG (Table 2, p<0.0001). While resection weight can only be definitively determined intra-operatively, many studies have proposed specific pre-operative measurements and equations to predict resection weight. Research has supported that a combination of horizontal and vertical measurements is the best way to accurately predict breast weight or volume [14-16]. The horizontal measurement measures the circumference of the breast along the IMF from the lateral aspect to the medial aspect near the sternum, while the vertical measurement begins at the IMF and ends above the breast meridian at the upper breast border [14]. Specifically, one study looked at different weight estimation formulas, including ones that utilized either BMI, vertical, sternal notch to nipple, horizontal, or horizontal × vertical measurements as the independent variable [15]. They found that the horizontal × vertical measurement was significantly correlated with the correct weight estimation [15]. While standard pre-operative measurements such as IMF to nipple and sternal notch to nipple are commonly done to predict pedicle viability, our study suggests that the most important predictor for performing an FNG is the weight of the resected specimen. Thus, pre-operative measurements that predict breast weight could possibly serve as more useful measurements. However, more studies are required to confirm this.

In addition to the weight of the resected specimens, patients with a higher pre-operative BMI were significantly associated with receiving a FNG (Table 1, p<0.0001). This was not surprising to us as obesity is generally correlated with higher rates of complications in both breast reduction and reconstructive procedures [17,18]. As a previous study has suggested, conversion to FNG should be considered in these heavier patients to avoid significant complications such as skin loss, wound dehiscence, and infection [13].

There are a few limitations to this study. It is important to note that while we had a large number of breast reduction patients, only 15 of the 323 patients (4.1%) received an FNG. However, given that the study was done at a large academic institution over a ten-year period and involved multiple experienced surgeons, this number also points towards how few FNG procedures are usually performed.

## Conclusions

In conclusion, this is the first study in over a decade to publish specific patient characteristics and indicators for the FNG procedure. According to our study, obese patients with resected weight specimens > 1500 g significantly indicate that an FNG should be performed. We believe that specific pre-operative measurements such as the IMF to nipple and sternal notch to nipple measurements do not necessarily indicate whether an FNG should be performed. Pre-operative weight estimating equations may be a viable option in predicting the need for an FNG, however, more studies would need to be done to confirm this.
